# Cost-Effectiveness of Pembrolizumab With Chemoradiotherapy for Locally Advanced Cervical Cancer

**DOI:** 10.1001/jamanetworkopen.2025.0033

**Published:** 2025-03-04

**Authors:** P. Travis Courtney, Puja S. Venkat, Ya-Chen Tina Shih, Albert J. Chang, Alan Lee, Michael L. Steinberg, Ann C. Raldow

**Affiliations:** 1 Department of Radiation Oncology, University of California, Los Angeles

## Abstract

**Importance:**

The KEYNOTE-A18 trial demonstrated that adding concurrent and adjuvant pembrolizumab to chemoradiotherapy and brachytherapy significantly improved survival in patients with newly diagnosed, locally advanced cervical cancer. However, considering the annual global incidence of 660 000 cases of cervical cancer, including 13 820 in the US in 2024, incorporating this regimen into the standard of care could have substantial health care economic implications for both patients and the health care system.

**Objective:**

To determine the cost-effectiveness of adding pembrolizumab to the first-line treatment of newly diagnosed, locally advanced cervical cancer.

**Design, Setting, and Participants:**

This economic evaluation created a Markov model simulating 50-year outcomes to evaluate cost-effectiveness from the payer perspective for patients receiving either pembrolizumab or placebo in addition to chemoradiotherapy plus brachytherapy. Probabilities, including disease progression, survival, and treatment-related toxic effects, were derived from KEYNOTE-A18 clinical trial data in patients with newly diagnosed, locally advanced cervical cancer. Costs and health utilities were obtained from published literature; 1-way, 3-way, and probabilistic sensitivity analyses were used to assess model uncertainty. Data analyses were conducted from April to November 2024.

**Exposure:**

Pembrolizumab.

**Main Outcomes and Measures:**

Costs, measured in 2024 US dollars, and effectiveness, measured in quality-adjusted life-years (QALYs) were used to calculate an incremental cost-effectiveness ratio (ICER). A willingness-to-pay threshold of $100 000 per QALY was chosen, below which pembrolizumab would be considered cost-effective.

**Results:**

KEYNOTE-A18 enrolled 1060 patients (529 in pembrolizumab group, 531 in placebo group). The median age was 50 years. Pembrolizumab increased costs by $257 000 and effectiveness by 1.40 QALYs, yielding an incremental cost-effectiveness ratio of $183 400 per QALY. The addition of pembrolizumab became cost-effective if its monthly cost was decreased from $16 990 to $9190 (a 45.6% reduction) or its maximum duration of 24 months was decreased to 10 months. The model was insensitive to assumptions about treatment-related toxic effects, progression-free survival, and overall survival. Probabilistic sensitivity analysis indicated that at a willingness-to-pay threshold of $100 000 per QALY, the addition of pembrolizumab was cost-effective 37.3% of the time.

**Conclusions and Relevance:**

In this economic evaluation of adding concurrent and adjuvant pembrolizumab to first-line treatment of newly diagnosed, locally advanced cervical cancer, this regimen was not cost-effective at current prices despite data demonstrating improved survival with this regimen.

## Key Points

**Question:**

Is the addition of concurrent and adjuvant pembrolizumab to first-line treatment with chemoradiotherapy and brachytherapy for newly diagnosed, locally advanced cervical cancer cost-effective from the US payer perspective?

**Findings:**

In this economic evaluation using data from the KEYNOTE-A18 randomized clinical trial, the addition of concurrent and adjuvant pembrolizumab increased costs by $257 000 and effectiveness by 1.40 quality-adjusted life-years (QALYs), yielding an incremental cost-effectiveness ratio of $183 400 per QALY.

**Meaning:**

This study suggests that at a willingness-to-pay threshold of $100 000 per QALY, the addition of concurrent and adjuvant pembrolizumab to first-line treatment of locally advanced cervical cancer is not cost-effective at current prices.

## Teaser

This economic evaluation investigates the cost-effectiveness of adding pembrolizumab to the first-line treatment of newly diagnosed, locally advanced cervical cancer.

## Introduction

Cervical cancer is the fourth most common cancer in people assigned female sex at birth worldwide with an estimated incidence of 660 000 cases in 2022 and 13 820 cases in the US in 2024.^1,2^ Moreover, cervical cancer–related mortality has stagnated or worsened in recent years, particularly in socioeconomically disadvantaged groups.^2,3^ First-line treatment of locally advanced cervical cancer has been chemoradiotherapy followed by brachytherapy^4,5^ for more than 2 decades despite suboptimal cure rates.^6,7,8,9^ The phase III ENGOT-cx11/GOG-3407/KEYNOTE-A18 randomized clinical trial evaluated the addition of concurrent and adjuvant pembrolizumab vs placebo to standard of care treatment in 1060 patients with newly diagnosed, locally advanced (International Federation of Gynecology and Obstetrics [FIGO] 2014 stages IB2-IVA) cervical cancer.^10,11^ The trial reported that pembrolizumab significantly improved progression-free and overall survival with an acceptable toxic effects profile. Improvements in the treatment of locally advanced cervical cancer are sorely needed, and thus there is substantial interest in incorporating this new regimen into the standard of care.^12^

Despite these benefits, immune checkpoints inhibitors (ICI) such as pembrolizumab are often associated with high costs^13,14,15^ which can negatively impact patients financially and aggravate financial toxicity.^16,17^ In cervical cancer specifically, the addition of pembrolizumab to chemotherapy was not found to be cost-effective for the treatment of persistent, recurrent, or metastatic disease in several cost-effectiveness studies,^18,19,20,21,22^ although 2 others did find this regimen to be cost-effective.^23,24^ However, the cost-effectiveness of pembrolizumab may differ in the frontline, definitive treatment setting. Health care economic concerns are distinctly relevant for cervical cancer not only because of its overall incidence, but also because of the well-established association between cervical cancer outcomes and patient socioeconomic factors.^3,25,26^ As such, we sought to analyze the cost-effectiveness of the addition of concurrent and adjuvant pembrolizumab vs placebo to chemoradiotherapy for the treatment of newly diagnosed, locally advanced cervical cancer.

## Methods

This cost-effectiveness analysis followed the Second Panel’s recommendations for the conducting, methodology, and reporting of cost-effectiveness analyses,^27^ as well as the Consolidated Health Economic Evaluation Reporting Standards (CHEERS) reporting guideline.^28^ An impact inventory is included as an eFigure in Supplement 1. This study included published clinical trial data without individual patient data and was therefore deemed exempt from review by the institutional review board of the University of California, Los Angeles.

### Decision Model

**Figure 1.**
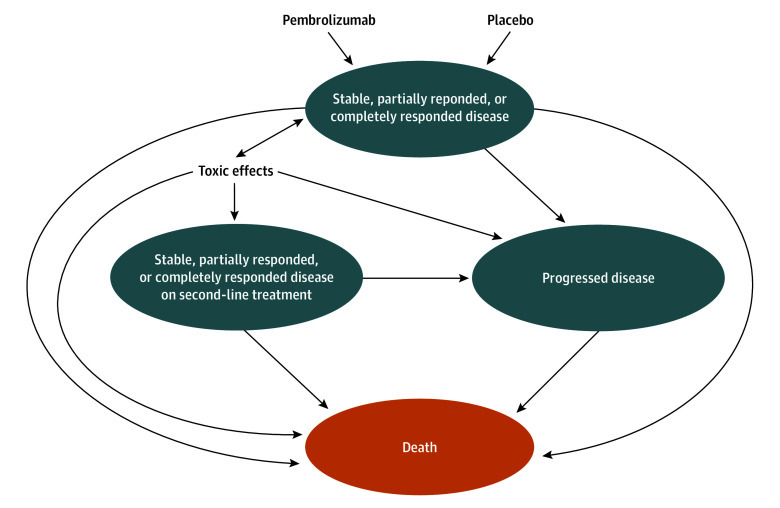
Health State Transition Diagram

We created a Markov model to analyze the cost-effectiveness of the addition of concurrent and adjuvant pembrolizumab to standard-of-care treatment of newly diagnosed, locally advanced cervical cancer. Model inputs of clinical parameters were derived from the KEYNOTE-A18 clinical trial.^10,11^ Patients could transition through 4 main health states: (1) stable, partially responded, or completely responded disease on first-line treatment; (2) stable, partially responded, or completely responded disease on second-line treatment; (3) progressive disease; or (4) death (Figure 1). Patients entered the model in the stable disease state and could continue having stable disease or develop partially or completely responded disease after the third month from completion of chemoradiotherapy, as this was when the first tumor imaging was performed per the KEYNOTE-A18 protocol. Patients could remain in this state or experience toxic effects, disease progression, or death. We assumed a hypothetical patient aged 50 years as this was the median age of KEYNOTE-A18 patients, and we used a 1-month cycle length with a half-cycle correction and a 50-year time horizon.

### Treatment Details

Our base case model followed the KEYNOTE-A18 protocol in which patients randomized to the experimental group received pembrolizumab 200 mg every 3 weeks for 5 cycles concurrently with chemoradiotherapy consisting of 5 cycles of weekly cisplatin 40 mg/m^2^ and external beam radiotherapy followed by brachytherapy. External beam radiotherapy was assumed to be 25 fractions and brachytherapy to be 5 fractions total with 5 individual implants. After 5 cycles of concurrent pembrolizumab, patients received pembrolizumab 400 mg every 6 weeks for up to 15 cycles. Therefore, the maximum duration of pembrolizumab was approximately 24 months. In our base model, patients continued first-line treatment for up to the median treatment duration reported by KEYNOTE-A18 (19 months pembrolizumab, 18 months placebo) or until disease progression or treatment-related toxic effects; however, not all patients in KEYNOTE-A18 who experienced treatment-related toxic effects discontinued first-line treatment. Therefore, patients who experienced first-line treatment-related toxic effects could either continue first-line treatment or switch to second-line treatment.

In both treatment groups, patients who experienced disease progression transitioned to second-line treatment. Additionally in our model, patients in the stable, partially responded, or completely responded disease state on second-line treatment who experienced disease progression were assumed to receive third-line treatment for the same duration as second-line treatment. The type or duration of subsequent-line treatments that patients received in KEYNOTE-A18 was not reported; therefore, our base model assumed that such patients received 10 monthly cycles of second-line treatment, which was the median treatment duration for the experimental group in the KEYNOTE-826 trial.^29^

### Model Probabilities

**Figure 2.**
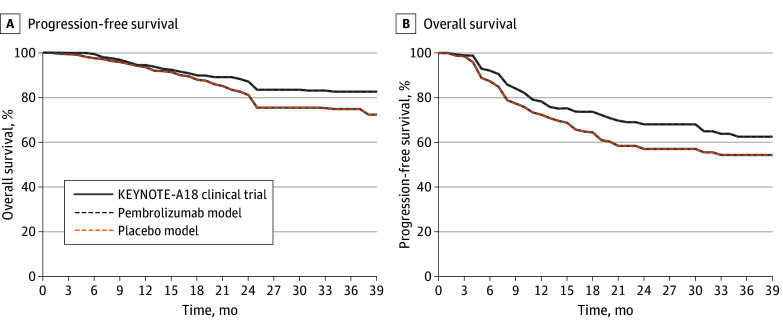
Model Validation This figure quantitatively compares progression-free (A) and overall survival (B) reported in the KEYNOTE-A18 trial with those produced by our model.

The transition probabilities for disease progression, death, and treatment-related toxic effects were derived from KEYNOTE-A18 data. Progression and survival data were extracted from Kaplan-Meier curves using a plot digitizer and converted to monthly transition probabilities.^30,31^ We included only grades 3 to 5 treatment-related adverse events as in prior cost-effectiveness studies.^30,32,33^ Projections of progression-free and overall survival by our model compared with the KEYNOTE-A18 trial is shown in Figure 2. The probability of discontinuing first-line treatment because of treatment-related toxic effects was 18.8% in the pembrolizumab group and 13.0% in the placebo group. The percentages of partial and complete responses were 24.6% and 63.0%, respectively, for the pembrolizumab group, and 27.2% and 56.5%, respectively, for the placebo group.

KEYNOTE-A18 survival data were reported through 39 months after randomization, after which we considered 2 assumptions for survival. In our base case, for patients alive after 39 months, monthly survival probability data were obtained from a cohort of patients with locally advanced cervical cancer in the Surveillance, Epidemiology, and End Results (SEER) database.^34^ SEER data were available through 18 years after diagnosis, at which point patient survival was assumed to follow general US population age-adjusted survival probabilities.^35^ In a sensitivity analysis, patients were considered cured of their cancer and their survival beyond 39 months followed the general US population age-adjusted survival probabilities.

### Other Model Inputs

Our base case considered costs from the health care payer perspective. We separately evaluated costs from a societal perspective which included parking, transportation, and meals during treatment,^36,37,38^ caregiver costs,^39^ and salary and/or productivity loss.^40^ Drug costs were calculated as the sum of the drug’s average wholesale price with a 7% reduction^32,41,42^ assuming a patient weight of 70 kg^43^ and a body surface area of 1.86 m^2^,^44^ costs of infusion,^45^ and converted to a monthly cost. External beam radiotherapy and brachytherapy costs were calculated from the Centers for Medicare and Medicaid Services Physician Fee Schedule,^45^ including the costs of consultation, simulation, and radiation planning, and assumed to be a one-time cost in the first (external beam radiotherapy portion) and second (brachytherapy portion) months. The types of subsequent-line treatments patients received in KEYNOTE-A18 were not reported. As such, monthly subsequent-line treatment costs were calculated as a mean of 2024 National Comprehensive Cancer Network guideline–endorsed subsequent-line treatments or best supportive care for cervical cancer,^4^ and are provided in eTable 1 in Supplement 1. Costs were adjusted to 2024 US dollars using the Consumer Price Index.^46^

Effectiveness was measured in quality-adjusted life years (QALYs), which is an aggregate measure of health utilities over time. Experiencing an event that may negatively impact health utility, such as disease progression or toxic effects, can be considered as a disutility (decrement). The health utilities used in this study were obtained from published literature. Treatment-related toxic effect costs and health disutilities were calculated as a weighted mean based on the reported treatment-related toxic effect frequencies for each group in the KEYNOTE-A18 trial. Individual toxic effect costs, including costs associated with clinical management, and disutilities were derived from the literature and are provided along with their references in eTables 2 and 3 in Supplement 1. The Table shows all model costs and health utilities with their respective literature sources.^19,20,36,37,38,39,40,41,42,45,47,48,49,50,51,52,53,54^ A 3% annual discount rate was applied to all costs and utilities.

**Table.** Base Case Model Inputs

Parameter	Value (95% CI)	Distribution, α (λ or β)^a^^,^^b^	Reference
**Costs per 1 month cycle, $**			
Pembrolizumab^c^	16 990 (10 860-24 417)	25 (λ: 0.001)	Average wholesale price^41^
Cisplatin^c^	170 (112-246)	25 (λ: 0.147)	Average wholesale price^42^
Second-line treatment	13 759 (9040-20 139)	25 (λ: 0.002)	Average wholesale price (eTable 1 in Supplement 1)
External beam radiotherapy^d^	22 687 (14 741-33 075)	25 (λ: 0.001)	CMS physician fee schedule^45^
Brachytherapy^e^	25 227 (16 818-36 654)	25 (λ: 0.001)	CMS physician fee schedule^45^
Stable disease	171 (112-248)	25 (λ: 0.146)	Mariotto et al,^47^ 2011
Progressed disease	495 (316-707)	25 (λ: 0.050)	Shi et al,^19^ 2022
Death^f^	11 572 (7530-16 259)	25 (λ: 0.002)	Shi et al,^19^ 2022
Toxic effects			
Pembrolizumab group^g^	13 612 (8438-18 705)	25 (λ: 0.002)	Wong et al,^48^ 2018; Insinga et al,^49^ 2019; Shi et al,^19^ 2022
Placebo group^g^	13 363 (8784-19 305)	25 (λ: 0.002)	Wong et al,^48^ 2018; Insinga et al,^49^ 2019; Shi et al,^19^ 2022
Parking, transportation, and meals during chemoradiotherapy	879 (571-1235)	25 (λ: 0.028)	Lauzier et al,^38^ 2011; Lee et al,^36^ 2020
Parking, transportation, and meals during systemic therapy only	77 (51-108)	25 (λ: 0.324)	Iragorri et al,^37^ 2021
Caregiver	747 (491-1090)	25 (λ: 0.033)	Li et al,^39^ 2013
Salary/productivity loss	440 (276-628)	25 (λ: 0.057)	Centers for Disease Control and Prevention,^40^ 2014
**Health utilities per year**			
Stable disease	0.736 (0.406-0.850)	1.35 (β: 20.7)	Sawaya et al,^50^ 2019
Partially responded disease	0.880 (0.521-0.930)	580 (β: 7608)	Gupta et al,^51^ 2022
Completely responded disease	0.909 (0.694-0.980)	370 (β: 4558)	Gupta et al,^51^ 2022
Disutility of being on active treatment	0.008 (0.005-0.012)	25.0 (β: 36 579)	Sawaya et al,^50^ 2019
Disutility of disease progression	0.038 (0.010-0.481)	0.201 (β: 63.2)	Shi et al,^19^ 2022
Toxic effects disutility			
Pembrolizumab group^h^	0.229 (0.146-0.324)	19.0 (β: 63.4)	Nafees et al,^52^ 2008; Houten et al,^53^ 2021; Li et al,^54^ 2022; Zheng et al,^20^ 2023
Placebo group^h^	0.221 (0.145-0.324)	19.0 (β: 63.7)	Nafees et al,^52^ 2008; Li et al,^54^ 2022; Zheng et al,^20^ 2023
Death	0	NA	NA

Abbreviations: CMS, Centers for Medicare & Medicaid Services; NA, not available.

^a^ For gamma distributions, α = 25 because an SD of 20% of the mean was used and the formula for α = ([mean value]^2^)/([0.2 × mean value]^2^).

^b^ λ for gamma distributions, β for beta distributions.

^c^ Includes costs of infusion ($129).^45^

^d^ Twenty-five fractions, weighted mean costs between intensity modulated radiotherapy/volumetric modulated arc therapy (89% [$22 999]) and 3-dimensional conformal radiotherapy (11% [$20 160]), per KEYNOTE-A18.

^e^ Five implants and/or 5 fractions.

^f^ Assumed as a 1-time cost over 1 month cycle.

^g^ Occurred over a 1-month cycle period. Calculated as a mean cost of toxic effects using weighted frequencies of grades 3 to 4 treatment-related toxic effects reported in the KEYNOTE-A18 clinical trial. Individual toxic effect costs, including costs associated with clinical management of the toxic effect, were derived from the literature and are provided along with their references in eTable 2 in Supplement 1.

^h^ Occurred over a 1-month cycle period. Calculated as a mean disutility of toxic effects using weighted frequencies of grades 3 to 4 treatment-related toxic effects reported in the KEYNOTE-A18 clinical trial. Individual toxic effect disutilities were derived from the literature and are provided along with their references in eTable 3 in Supplement 1.

### Statistical Analysis

The primary end point was incremental cost-effectiveness ratio (ICER), which is the ratio of differences in mean costs between the pembrolizumab and placebo groups, measured in 2024 US dollars, divided by difference in mean effectiveness, measured in QALYs. We used a willingness-to-pay (WTP) threshold of $100 000 per QALY, below which the addition of concurrent and adjuvant pembrolizumab would be considered cost-effective compared with current standard of care.^55,56^ To evaluate the effect of individual model inputs on the ICER, we performed 1-way sensitivity analyses of each input parameter. Given the interrelatedness between the probabilities of different responses to treatment (ie, as the percentage of complete responders increase, the percentages of partially and stable responders decrease), a 3-way sensitivity analysis of these inputs was performed. We further assessed model uncertainty with a probabilistic sensitivity analysis using a Monte Carlo simulation with 100 000 samples simultaneously varying model inputs over their distribution range. Gamma distributions were used for costs, and β distributions were used for health utilities and transition probabilities; 95% CIs for distributions were obtained from published literature, though in cases when SDs were unknown, they were calculated to be 20% of the mean. Varying the unknown SD between 10% and 40% of the mean did not meaningfully change our results (eTable 4 in Supplement 1). The model was created and analyzed with TreeAge Pro Healthcare 2024. Data analyses were conducted from April to November 2024.

## Results

### Base Case Analysis

Among the 1060 patients (529 in pembrolizumab group, 531 in placebo group) enrolled in the KEYNOTE-A18 trial at 176 sites in 30 countries across Asia, Australia, Europe, North America, and South America, the median age was 50 years. Our base case analysis found that the addition of concurrent and adjuvant pembrolizumab increased overall costs by $257 000, from $106 200 in the placebo group to $363 200 in the pembrolizumab group, and increased overall effectiveness by 1.40 QALYs, from 10.50 in the placebo group to 11.90 in the pembrolizumab group. This resulted in an ICER of $183 400 per QALY, which was not considered cost-effective at a WTP threshold of $100 000 per QALY. From a societal perspective, the model yielded an ICER of $199 100 per QALY.

### One- and Three-Way Sensitivity Analyses

**Figure 3.**
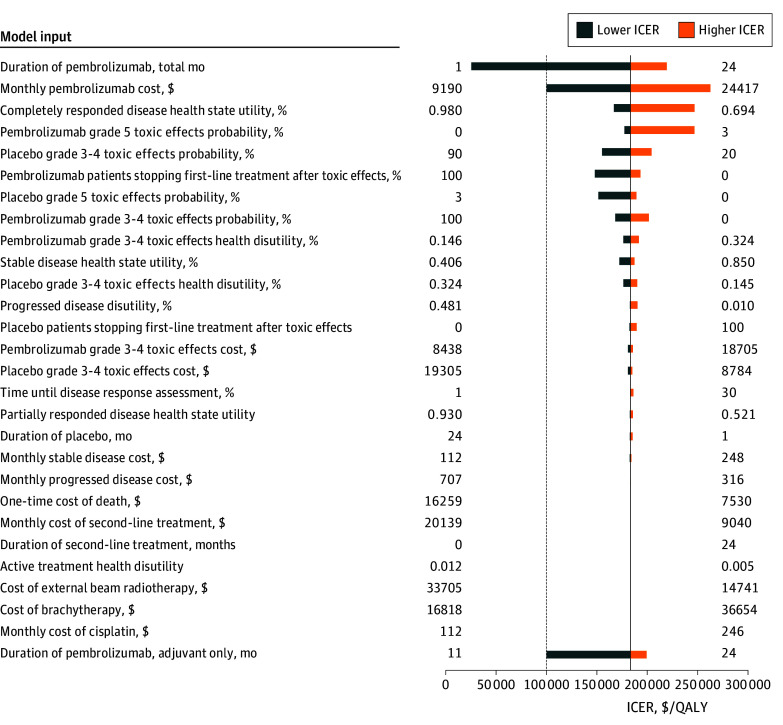
Tornado Diagram of 1-Way Sensitivity Analyses Including Break-Even Analyses for Pembrolizumab Cost, Total Duration, and Duration in the Adjuvant-Only Setting The vertical dashed line represents the $100 000 per quality-adjusted life-year (QALY) willingness-to-pay threshold. ICER indicates incremental cost-effectiveness ratio.

One-way sensitivity analyses revealed our base model to be moderately sensitive to the cost and duration of pembrolizumab. When testing model sensitivity to pembrolizumab duration or timing, we assumed that all toxic effects and outcome differences associated with pembrolizumab compared with placebo remained the same as reported by KEYNOTE-A18. The monthly cost of pembrolizumab or maximum allowable duration would have to decrease from $16 990 to $9190 (a 45.6% reduction) or from 24 months to 10 months, respectively, to become cost-effective at a WTP threshold of $100 000 per QALY. If we assumed that pembrolizumab was only given concurrent with chemoradiotherapy for 5 triweekly cycles, or approximately 4 months total, per KEYNOTE-A18 protocol, the ICER decreased to $50 000 per QALY. If we assumed that pembrolizumab was only given adjuvantly after completion of chemoradiotherapy, the maximum allowable duration of pembrolizumab for which this strategy would remain cost-effective was 11 months. Figure 3 includes the results of these break-even analyses.

The model was not sensitive to other costs, utilities, or treatment response or toxic effect probabilities. Model sensitivity to various inputs is summarized in Figure 3. On 3-way analysis of pembrolizumab or placebo response probabilities, there were no combinations of response to pembrolizumab which yielded an ICER less than $100 000 per QALY, while only combinations in which the probability of stable disease after placebo was at least 80% (5 of 66 combinations) yielded an ICER less than $100 000 per QALY (eTables 5 and 6 in Supplement 1).

Our model was modestly sensitive to assumptions about survival. KEYNOTE-A18 reported a hazard ratio (HR) of 0.67 (95% CI, 0.50-0.90) for death for patients receiving pembrolizumab. Decreasing the HR below 0.56 lowered the ICER below the $100 000 per QALY WTP threshold. When we assumed all patients alive beyond the trial reported range were cured rather than having followed SEER survival probabilities, the ICER decreased to $157 900 per QALY.

### Probabilistic Sensitivity Analyses

**Figure 4.**
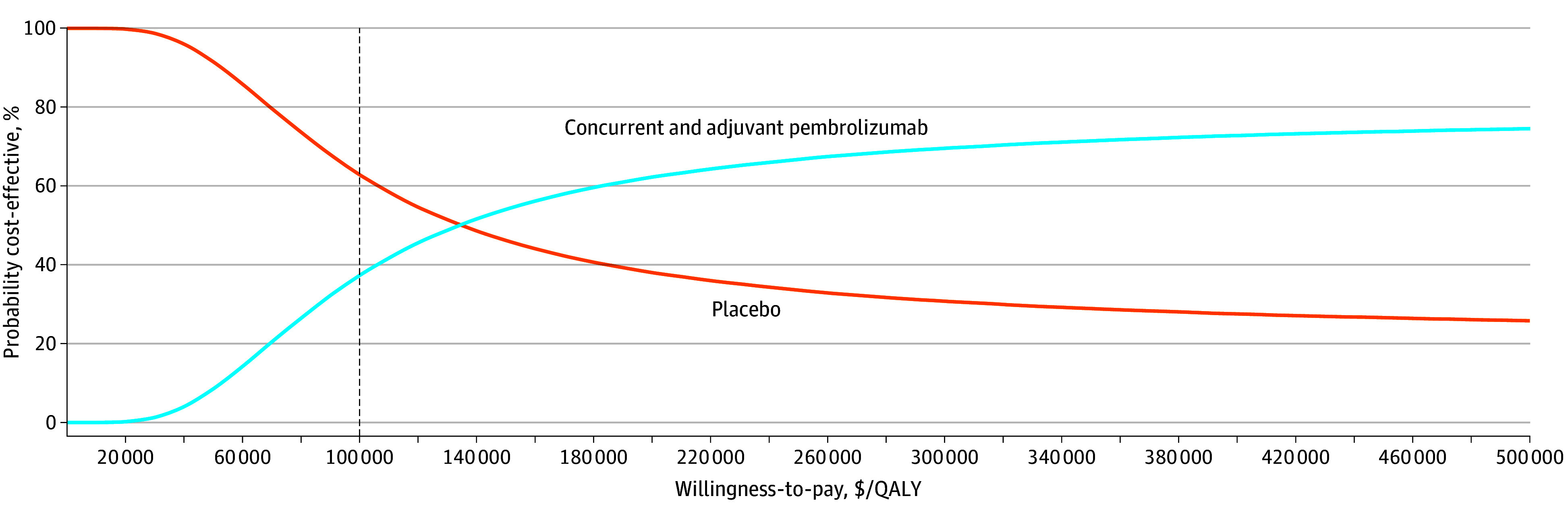
Probabilistic Sensitivity Analysis The vertical dashed line represents the $100 000 per quality-adjusted life year (QALY) willingness-to-pay threshold.

Probabilistic sensitivity analysis found that at a WTP threshold of $100 000 per QALY, the probability that concurrent and adjuvant pembrolizumab would be the cost-effective option was 37.3% (Figure 4). If we increased the WTP threshold to $150 000 per QALY, the probability that pembrolizumab would be the cost-effective option increased to 53.9%.

## Discussion

In this cost-effectiveness analysis of the addition of concurrent and adjuvant pembrolizumab to standard of care treatment of newly diagnosed, locally advanced cervical cancer, we found that this pembrolizumab-based regimen would not be considered cost-effective at current prices. Our model was not sensitive to numerous model inputs, including current pembrolizumab price and maximum duration of pembrolizumab. To become cost-effective, the drug price would have to be decreased by 45.6% (from $16 990 to $9190 per month) or the maximum pembrolizumab duration decreased to 10 months. To our knowledge, no manuscript published to date has evaluated the cost-effectiveness of pembrolizumab or any other ICI regimen in the first-line, definitive treatment of localized cervical cancer.

The optimal ICI duration is an ongoing subject of study, and it is relevant to the current study as duration often impacts cost-effectiveness given the high costs of ICIs.^32,57^ In locally advanced cervical cancer, both the KEYNOTE-A18 trial and CALLA trial,^58^ which evaluated the addition of durvalumab to chemoradiotherapy and brachytherapy for locally advanced cervical cancer, gave concurrent and adjuvant ICI. In contrast to KEYNOTE-A18, CALLA did not detect a significant difference in progression-free survival between the durvalumab and placebo groups. This discordance may be attributed to the different molecular targets of pembrolizumab vs durvalumab (PD-1 on cytotoxic T-cells vs PD-L1 on tumor cells, respectively) or differences in study populations,^59^ although it is unlikely that the different maximum cycles of ICI permitted in the KEYNOTE-A18 and CALLA trials (20 and 24, respectively) would produce the contrasting results, especially because the median ICI durations in each study were similar.

For pembrolizumab specifically, outcomes by duration of ICI were not reported in the KEYNOTE-826 or KEYNOTE-A18 trials. This topic has been evaluated in other cancers as well, such as non–small-cell lung cancer, melanoma, and renal cell carcinoma, with variable results; although in general it has been suggested to be on the order of months to years.^60,61,62^ Notably, the majority of the data on ICI duration are in patients with recurrent or metastatic disease. Considerations surrounding ICI duration may differ for patients with newly diagnosed, localized disease, such as those in KEYNOTE-A18, as prolonged treatment with ICIs may not be necessary for this population with a lower overall cancer burden and/or less aggressive disease phenotype. While the current study found that decreasing the duration of pembrolizumab is an avenue to improve its cost-effectiveness, this is under the assumption that doing so does not change its toxic effects and outcomes, which is currently an unanswered question. Ultimately, optimization of pembrolizumab duration may improve its cost-effectiveness as part of the first-line treatment of locally advanced cervical cancer.

There is also uncertainty surrounding the optimal sequencing of ICI with radiotherapy in cervical and other cancers,^63,64^ including the similarly human papillomavirus-associated head and neck cancer.^65,66,67^ In general, trials of ICIs given concurrently with (chemo)radiotherapy have not met their primary outcome end points.^68^ When given after radiotherapy with or without surgery and/or chemotherapy, ICIs have demonstrated clinical benefits, and subsequently, cost-effectiveness, in early-stage triple-negative breast cancer,^69^ non–small-cell lung cancer,^70^ and esophageal cancer.^71,72^ In this cost-effectiveness study, we found that giving pembrolizumab only in the adjuvant setting for a maximum of 11 months was a cost-effective strategy. Additionally, giving pembrolizumab only concurrently with chemoradiotherapy yielded an ICER of $50 000 per QALY, below the $100 000 per QALY WTP threshold. Again, in both of these scenarios, we assumed constant toxic effects and outcomes data as published. This suggests that treatment with either concurrent or adjuvant pembrolizumab alone in addition to standard chemoradiotherapy may be a mechanism by which pembrolizumab can become cost-effective in this clinical context, provided those alternative regimens are found to be clinically safe and efficacious.

This study highlights the persistent concerns surrounding the high costs associated with ICIs,^73^ which are even more pertinent in cervical cancer. Disparities in cervical cancer incidence and mortality across racial, ethnic, and income groups^3,26,74,75^ and by insurance status^76,77^ are well-documented and exist both within the US and worldwide.^2,78^ These expensive treatments may ultimately be detrimental to these disadvantaged groups and exacerbate existing disparities as the result of financial toxicity or inability to afford the most clinically beneficial drugs. Although the US Food and Drug Administration (FDA) recently approved pembrolizumab with chemoradiotherapy and brachytherapy for the treatment of FIGO 2014 stage III-IVA cervical cancer,^79^ FDA approval of a drug does not mandate insurance coverage.^80^ Even with insurance coverage, a large proportion of patients with cervical cancer are underinsured or uninsured^81,82^ and therefore may still be subject to high out-of-pocket costs. If this pembrolizumab-based regimen assessed in our study is to be implemented into the standard of care for locally advanced cervical cancer, care must be taken so as to not financially harm an already at-risk population.

### Limitations

This study had limitations. We constructed our model predominantly using data from the first and second interim reports of a single clinical trial. This is an early economic assessment of this regimen, and longer-term follow-up may reveal new data that might impact the cost-effectiveness of this regimen,^83^ such as further improvement in the overall survival benefit associated with pembrolizumab at publication of the final analysis. Specific toxic effect frequencies and monthly transition probabilities for each patient subgroup were not reported, and therefore the cost-effectiveness of this treatment in these subgroups may differ in future analyses if these additional data become available. More broadly, costs, response to treatment, and health-related quality of life can vary considerably across individual patients or health care systems, and these results may differ for patients or health care settings outside of the KEYNOTE-A18 trial.^83^ As in other cost-effectiveness analyses, we obtained costs and health utility inputs from multiple outside sources as the current publications of KEYNOTE-A18 trial do not specify nor provide results from an accompanying prospective economic analysis,^84,85,86^ though in general our model was relatively insensitive to these inputs.

## Conclusions

This economic evaluation found that the addition of concurrent and adjuvant pembrolizumab to chemoradiotherapy for newly diagnosed, locally advanced cervical cancer was not cost-effective despite its clinical benefits. While this pembrolizumab-based regimen is promising and much needed in this patient population, one should remain cognizant of its high costs and the subsequent impact this may have on patients and the health care system as a whole.

## Notes

Supplement 1. **eFigure.** Second Panel Impact Inventory **eTable 1.** Base Case Model Second-Line Treatment Costs **eTable 2.** Associated Costs of Grade 3-4 Treatment-Related Adverse Events **eTable 3.** Disutility From Grade 3-4 Treatment-Related Adverse Events **eTable 4.** Unknown Standard Deviation Sensitivity Analysis **eTable 5.** Three-Way Sensitivity Analysis of Disease Response to Pembrolizumab **eTable 6.** Three-Way Sensitivity Analysis of Disease Response to Placebo

Supplement 2.
Data Sharing Statement
