# Supplementary material for: Cost-Effectiveness of Pembrolizumab With Chemoradiotherapy for Locally Advanced Cervical Cancer
Source: JAMA Netw Open. 2025 Mar 4;8(3):e250033. doi: 10.1001/jamanetworkopen.2025.0033 (PMC11880949; doi:10.1001/jamanetworkopen.2025.0033)
Supplement: Supplement 1. — eFigure. Second Panel Impact Inventory eTable 1. Base Case Model Second-Line Treatment Costs eTable 2. Associated Costs of Grade 3-4 Treatment-Related Adverse Events eTable 3. Disutility From Grade 3-4 Treatment-Related Adverse Events eTable 4. Unknown Standard Deviation Sensitivity Analysis eTable 5. Three-Way Sensitivity Analysis of Disease Response to Pembrolizumab eTable 6. Three-Way Sensitivity Analysis of Disease Response to Placebo [file jamanetwopen-e250033-s001.pdf]

Supplemental Online Content

Courtney PT, Venkat PS, Shih YT, et al. Cost-effectiveness of pembrolizumab with chemoradiotherapy for locally advanced cervical cancer. *JAMA Netw Open.* 2025;8(3):e250033. doi:10.1001/jamanetworkopen.2025.0033

eFigure. Second Panel Impact Inventory

eTable 1. Base Case Model Second-Line Treatment Costs

eTable 2. Associated Costs of Grade 3-4 Treatment-Related Adverse Events

eTable 3. Disutility From Grade 3-4 Treatment-Related Adverse Events

eTable 4. Unknown Standard Deviation Sensitivity Analysis

eTable 5. Three-Way Sensitivity Analysis of Disease Response to Pembrolizumab

eTable 6. Three-Way Sensitivity Analysis of Disease Response to Placebo

This supplemental material has been provided by the authors to give readers additional information about their work.

© 2025 Courtney PT et al. *JAMA Network Open*.

eFigure. Second Panel Impact Inventory

Sector	Type of Impact (list category within each sector with unit of measure if relevant)[a]	Included in This Reference Case Analysis From...Perspective?		Notes on Sources of Evidence
		Health Care Sector	Societal	
**Formal Health Care Sector**				
Health	Health outcomes (effects)			
	Longevity effects	X	X	
	Health-related quality-of-life effects	X	X	
	Other health effects (eg, adverse events and secondary transmissions of infections)	X	X	
	Medical costs			
	Paid for by third-party payers	X	X	
	Paid for by patients out-of-pocket	X	X	
	Future related medical costs (payers and patients)	X	X	
	Future unrelated medical costs (payers and patients)	X	X	
**Informal Health Care Sector**				
Health	Patient-time costs	NA	☐	
	Unpaid caregiver-time costs	NA	X	
	Transportation costs	NA	X	
**Non–Health Care Sectors (with examples of possible items)**				
Productivity	Labor market earnings lost	NA	X	
	Cost of unpaid lost productivity due to illness	NA	X	
	Cost of uncompensated household production[b]	NA	☐	
Consumption	Future consumption unrelated to health	NA	☐	
Social Services	Cost of social services as part of intervention	NA	☐	
Legal or Criminal Justice	Number of crimes related to intervention	NA	☐	
	Cost of crimes related to intervention	NA	☐	
Education	Impact of intervention on educational achievement of population	NA	☐	
Housing	Cost of intervention on home improvements (eg, removing lead paint)	NA	☐	
Environment	Production of toxic waste pollution by intervention	NA	☐	
Other (specify)	Other impacts	NA	☐	

© 2025 Courtney PT et al. *JAMA Network Open*.

eTable 1: Base case model second-line treatment costs. References below.

Treatment	Estimated Monthly Cost ($)	Reference
Bevacizumab	$2,679	Average Wholesale Price[1]
Carboplatin	$335	Average Wholesale Price[2]
Cemiplimab	$16,649	Average Wholesale Price[3]
Cisplatin	$170	Average Wholesale Price[4]
Docetaxel	$2,415	Average Wholesale Price[5]
Entrectinib	$24,052	Average Wholesale Price[6]
Fam-trastuzumab deruxtecan-nxk	$17,464	Average Wholesale Price[7]
Fluorouracil	$335	Average Wholesale Price[8]
Gemcitabine	$369	Average Wholesale Price[9]
Irinotecan	$177	Average Wholesale Price[10]
Larotrectinib	$46,782	Average Wholesale Price[11]
Nivolumab	$18,222	Average Wholesale Price[12]
Paclitaxel	$393	Average Wholesale Price[13]
Pembrolizumab	$16,990	Average Wholesale Price[14]
Pemetrexed	$12,956	Average Wholesale Price[15]
Selpercatinib	$27,118	Average Wholesale Price[16]
Tisotumab	$44,051	Average Wholesale Price[17]
Topotecan	$574	Average Wholesale Price[18]
Vinorelbine	$992	Average Wholesale Price[19]
Brachytherapy, 5 fractions/5 implants	$25,227	CMS Physician Fee Schedule[19]
Intensity Modulated Radiotherapy, 5 fractions	$9,994	CMS Physician Fee Schedule[19]
Pelvic Exenteration	$66,316	PelxEx Collaborative,[20] 2020
Best Supportive Care	$4,954	Shi et al,[21] 2022
Base Case Model Input[a,b]	***$13,759***	

[a]Drug costs were calculated as the sum of the drug's average wholesale price assuming a patient weight of 70 kg[22] and a body surface area of 1.86 m^2 [23]; costs of infusion[19]; a 7% reduction[24]; and converted to a monthly cost.

[b]Average of all 2024 National Comprehensive Cancer Network guideline-endorsed subsequent-line treatments[25].

References

1. UpToDate. Bevacizumab (including biosimilars): Drug information. https://www.uptodate.com/contents/bevacizumab-including-biosimilars-drug-information

2. UpToDate. Carboplatin: Drug information. https://www.uptodate.com/contents/carboplatin-drug-information

3. UpToDate. Cemiplimab: Drug information. https://www.uptodate.com/contents/cemiplimab-drug-information

4. UpToDate. Cisplatin: Drug information. https://www.uptodate.com/contents/cisplatin-drug-information

5. UpToDate. Docetaxel: Drug information. https://www.uptodate.com/contents/docetaxel-drug-information

6. UpToDate. Entrectinib: Drug information. https://www.uptodate.com/contents/entrectinib-drug-information

7. UpToDate. Fam-trastuzumab deruxtecan: Drug information. https://www.uptodate.com/contents/fam-trastuzumab-deruxtecan-drug-information

8. UpToDate. Fluorouracil (systemic): Drug information. https://www.uptodate.com/contents/fluorouracil-systemic-drug-information

9. UpToDate. Gemcitabine: Drug information. https://www.uptodate.com/contents/gemcitabine-drug-information

10. UpToDate. Irinotecan (conventional): Drug information. https://www.uptodate.com/contents/irinotecan-conventional-drug-information

11. UpToDate. Larotrectinib: Drug information. https://www.uptodate.com/contents/larotrectinib-drug-information

12. UpToDate. Nivolumab: Drug information. https://www.uptodate.com/contents/nivolumab-drug-information

13. UpToDate. Paclitaxel (conventional): Drug information. https://www.uptodate.com/contents/paclitaxel-conventional-drug-information

14. UpToDate. Pembrolizumab: Drug information. https://www.uptodate.com/contents/pembrolizumab-drug-information

15. UpToDate. Pemetrexed: Drug information. https://www.uptodate.com/contents/pemetrexed-drug-information

16. UpToDate. Selpercatinib: Drug information. https://www.uptodate.com/contents/selpercatinib-drug-information

17. UpToDate. Tisotumab vedotin: Drug information. https://www.uptodate.com/contents/tisotumab-vedotin-drug-information

18. UpToDate. Topotecan: Drug information. https://www.uptodate.com/contents/topotecan-drug-information

19. Centers for Medicare & Medicaid Services. Search the Physician Fee Schedule. https://www.cms.gov/medicare/physician-fee-schedule/search

20. PelvEx Collaborative. The global cost of pelvic exenteration: in-hospital perioperative costs. *Br J Surg*. 2020;107(11):e470-e471. doi:10.1002/bjs.11924

21. Shi Y, Chen J, Shi B, Liu A. Cost-effectiveness analysis of pembrolizumab for treatment of US patients with persistent, recurrent, or metastatic cervical cancer. *Gynecol Oncol*. 2022;164(2):379-385. doi:10.1016/j.ygyno.2021.12.007

© 2025 Courtney PT et al. *JAMA Network Open*.

22. Cortellini A, Bersanelli M, Buti S, et al. A multicenter study of body mass index in cancer patients treated with anti-PD-1/PD-L1 immune checkpoint inhibitors: when overweight becomes favorable. *J Immunother Cancer*. 2019;7(1):57. doi:10.1186/s40425-019-0527-y

23. Baker SD, Verweij J, Rowinsky EK, et al. Role of body surface area in dosing of investigational anticancer agents in adults, 1991-2001. *J Natl Cancer Inst*. 2002;94(24):1883-1888. doi:10.1093/jnci/94.24.1883

24. Courtney PT, Yip AT, Cherry DR, Salans MA, Kumar A, Murphy JD. Cost-effectiveness of Nivolumab-Ipilimumab Combination Therapy for the Treatment of Advanced Non-Small Cell Lung Cancer. *JAMA Netw Open*. 2021;4(5):e218787. doi:10.1001/jamanetworkopen.2021.8787

25. National Comprehensive Cancer Network. *Cervical Cancer Version 3.2024*.; 2024. https://www.nccn.org/professionals/physician_gls/pdf/cervical.pdf

© 2025 Courtney PT et al. *JAMA Network Open*.

eTable 2. Associated costs of grade 3-4 treatment-related adverse events. References per main text.

**Adverse Event**[a]	**No. of patients (%)**[b]	**Costs in 2024 USD**[c]	**Reference**
**Pembrolizumab**			
White blood cell count decreased, leukopenia	174 (32.9%)	$21,552	Wong et al,[43] 2018
Anemia	122 (23.1%)	$14,432	Shi et al,[17] 2022
Neutrophil count decreased	83 (15.7%)	$1,138	Insinga et al,[44] 2019
Neutropenia	61 (11.6%)	$10,455	Shi et al,[17] 2022
Diarrhea	27 (5.1%)	$4,256	Shi et al,[17] 2022
Platelet count decreased	25 (4.7%)	$12,711	Insinga et al,[44] 2019
Nausea, vomiting	13 (2.5%)	$4,267	Shi et al,[17] 2022
Hypo/hyperthyroidism	5 (0.9%)	$25,625	Wong et al,[43] 2018
Weighted average[d]	-	*$13,499*	
**Placebo**			
White blood cell count decreased, leukopenia	172 (32.4%)	$21,552	Wong et al,[43] 2018
Anemia	117 (22.1%)	$14,432	Shi et al,[17] 2022
Neutrophil count decreased	81 (15.3%)	$1,138	Insinga et al,[44] 2019
Neutropenia	57 (10.8%)	$10,455	Shi et al,[17] 2022
Diarrhea	25 (4.7%)	$4,256	Shi et al,[17] 2022
Nausea, vomiting	17 (3.2%)	$4,267	Shi et al,[17] 2022
Platelet count decreased	15 (2.8%)	$12,711	Insinga et al,[44] 2019
Weighted average[d]	-	*$13,332*	

[a]Refers to treatment-related adverse events of any grade that occurred in ≥15% of total patients in the KEYNOTE-A18 trial. Our analysis only included and evaluated grade 3-4 treatment-related adverse events.

[b]Number within each treatment arm: pembrolizumab (N=528), placebo (N=530).

[c]Cost per one-month cycle.

[d]Calculated as an average cost of toxicity using the weighted frequency of occurrence. This value was used in the base-case model.

© 2025 Courtney PT et al. *JAMA Network Open*.

eTable 3. Disutility from grade 3-4 treatment-related adverse events. References per main text.

**Adverse Event**[a]	**No. of patients (%)**[b]	**Disutility**[c]	**Reference**
**Pembrolizumab**			
White blood cell count decreased, leukopenia	174 (32.9%)	0.20	Li et al,[49] 2022
Anemia	122 (23.1%)	0.38	Zheng et al,[18] 2023
Neutrophil count decreased	83 (15.7%)	0.20	Zheng et al,[18] 2023
Neutropenia	61 (11.6%)	0.20	Zheng et al,[18] 2023
Platelet count decreased	27 (5.1%)	0.27	Zheng et al,[18] 2023
Diarrhea	25 (4.7%)	0.018	Nafees et al,[47] 2008;
Nausea, vomiting	13 (2.5%)	0.017	Nafees et al,[47] 2008;
Hypo/hyperthyroidism	5 (0.9%)	0.042	Houten et al,[48] 2021
Weighted average[d]	-	*0.231*	
**Placebo**			
White blood cell count decreased, leukopenia	172 (32.4%)	0.20	Li et al,[49] 2022
Anemia	117 (22.1%)	0.38	Zheng et al,[18] 2023
Neutrophil count decreased	81 (15.3%)	0.20	Zheng et al,[18] 2023
Neutropenia	57 (10.8%)	0.20	Zheng et al,[18] 2023
Diarrhea	25 (4.7%)	0.018	Nafees et al,[47] 2008;
Nausea, vomiting	17 (3.2%)	0.017	Nafees et al,[47] 2008;
Platelet count decreased	15 (2.8%)	0.27	Zheng et al,[18] 2023
Weighted average[d]	-	*0.230*	

[a]Refers to treatment-related adverse events of any grade that occurred in ≥15% of total patients in the KEYNOTE-A18 trial. Our analysis only included and evaluated grade 3-4 treatment-related adverse events.

[b]Number within each treatment arm: pembrolizumab (N=528), placebo (N=530).

[c]Disutility per one-month cycle.

[d]Calculated as an average disutility of toxicity using the weighted frequency of occurrence. This value was used in the base-case model.

© 2025 Courtney PT et al. *JAMA Network Open*.

eTable 4: Unknown standard deviation sensitivity analysis.

Unknown Standard Deviation	Probability of Pembrolizumab Being Cost-Effective at $100,000/QALY	Probability of Pembrolizumab Being Cost-Effective at $150,000/QALY
10%	35.3%	56.1%
20%[a]	37.3%	53.9%
30%	38.9%	52.8%
40%	40.6%	51.9%

[a]This value was used in the base-case model.

© 2025 Courtney PT et al. *JAMA Network Open*.

eTable 5: Three-way sensitivity analysis of disease response to pembrolizumab.

Probability of complete response with pembrolizumab	Probability of partial response with pembrolizumab	Probability of stable disease with pembrolizumab	ICER ($/QALY)
10%	10%	80%	-12967145.98
0%	20%	80%	-2924603.86
10%	0%	90%	-1452430.32
0%	10%	90%	-1048976.87
0%	0%	100%	-639102.97
100%	0%	0%	138942.42
90%	10%	0%	144249.83
80%	20%	0%	149978.81
70%	30%	0%	156181.68
90%	0%	10%	158201.97
60%	40%	0%	162919.75
80%	10%	10%	165119.35
50%	50%	0%	170265.44
70%	20%	10%	172669.33
40%	60%	0%	178304.81
60%	30%	10%	180942.82
80%	0%	20%	183660.06
30%	70%	0%	187140.98
50%	40%	10%	190049.06
70%	10%	20%	193048.95
20%	80%	0%	196898.59
40%	50%	10%	200120.45
60%	20%	20%	203449.50
10%	90%	0%	207729.72
30%	60%	10%	211319.01
50%	30%	20%	215034.52
70%	0%	30%	218883.03
0%	100%	0%	219821.81
20%	70%	10%	223845.19
40%	40%	20%	228018.59
60%	10%	30%	232350.56
10%	80%	10%	237949.95
30%	50%	20%	242671.40
50%	20%	30%	247584.03
0%	90%	10%	253951.75
20%	60%	20%	259336.77

© 2025 Courtney PT et al. *JAMA Network Open*.

40%	30%	30%	264955.12
60%	0%	40%	270822.29
10%	70%	20%	278459.90
30%	40%	30%	284947.74
50%	10%	40%	291745.11
0%	80%	20%	300627.77
20%	50%	30%	308203.76
40%	20%	40%	316171.45
10%	60%	30%	335593.21
30%	30%	40%	345061.72
50%	0%	50%	355080.05
0%	70%	30%	368325.56
20%	40%	40%	379762.67
40%	10%	50%	391932.82
10%	50%	40%	422223.32
30%	20%	50%	437321.16
0%	60%	40%	475374.14
20%	30%	50%	494598.89
40%	0%	60%	515444.13
10%	40%	50%	569141.70
30%	10%	60%	596920.20
0%	50%	50%	670141.13
20%	20%	60%	708990.00
10%	30%	60%	872868.11
30%	0%	70%	939953.34
0%	40%	60%	1135280.25
20%	10%	70%	1251448.68
10%	20%	70%	1871729.47
0%	30%	70%	3711173.27
20%	0%	80%	5327914.34

© 2025 Courtney PT et al. *JAMA Network Open*.

eTable 6: Three-way sensitivity analysis of disease response to placebo.

Probability of complete response with placebo	Probability of partial response with placebo	Probability of stable disease with placebo	ICER ($/QALY)
0%	0%	100%	88298.20836
0%	10%	90%	92742.41742
10%	0%	90%	94809.28261
0%	20%	80%	97657.70704
10%	10%	80%	99952.17312
20%	0%	80%	102357.0502
0%	30%	70%	103123.17
10%	20%	70%	105685.0132
20%	10%	70%	108377.3844
0%	40%	60%	109236.6546
30%	0%	70%	111210.5204
10%	30%	60%	112115.4904
20%	20%	60%	115150.1717
0%	50%	50%	116120.6745
30%	10%	60%	118353.7056
10%	40%	50%	119379.1998
40%	0%	60%	121740.5881
20%	30%	50%	122825.8835
0%	60%	40%	123930.7034
30%	20%	50%	126477.5077
10%	50%	40%	127649.3103
40%	10%	50%	130352.9111
20%	40%	40%	131597.9778
0%	70%	30%	132867.0584
50%	0%	50%	134473.3143
30%	30%	40%	135798.7374
10%	60%	30%	137150.5483
40%	20%	40%	140276.5262
20%	50%	30%	141719.429
0%	80%	20%	143192.3241
50%	10%	40%	145059.6829
30%	40%	30%	146603.205
10%	70%	20%	148179.9284
60%	0%	40%	150180.5474
40%	30%	30%	151835.5929
20%	60%	20%	153527.5234
0%	90%	10%	155257.5857

© 2025 Courtney PT et al. *JAMA Network Open*.

50%	20%	30%	157455.301
30%	50%	20%	159275.5426
10%	80%	10%	161138.3619
60%	10%	30%	163506.9883
40%	40%	20%	165470.7109
20%	70%	10%	167482.1755
0%	100%	0%	169543.1447
70%	0%	30%	170042.4544
50%	30%	20%	172167.3165
30%	60%	10%	174345.9554
10%	90%	0%	176580.4388
60%	20%	20%	179428.8067
40%	50%	10%	181796.3614
20%	80%	0%	184227.2311
70%	10%	20%	187329.8042
50%	40%	10%	189911.9566
30%	70%	0%	192566.2885
80%	0%	20%	195958.681
60%	30%	10%	198785.9878
40%	60%	0%	201696.0742
70%	20%	10%	208529.9852
50%	50%	0%	211734.6538
80%	10%	10%	219278.4736
60%	40%	0%	222824.8289
90%	0%	10%	231195.2209
70%	30%	0%	235140.9733
80%	20%	0%	248898.2688
90%	10%	0%	264365.3849
100%	0%	0%	281882.1989

© 2025 Courtney PT et al. *JAMA Network Open*.
