# Supplementary material for: Cost-Effectiveness of Pembrolizumab With Chemoradiotherapy for Locally Advanced Cervical Cancer
Source: JAMA Netw Open. 2025 Mar 4;8(3):e250033. doi: 10.1001/jamanetworkopen.2025.0033 (PMC11880949; doi:10.1001/jamanetworkopen.2025.0033)
Supplement: Supplement 2. — Data Sharing Statement [file jamanetwopen-e250033-s002.pdf]

Data Sharing Statement

Courtney. Cost-Effectiveness of Pembrolizumab With Chemoradiotherapy for Locally Advanced Cervical Cancer. *JAMA Netw Open*. Published March 04, 2025. doi:10.1001/jamanetworkopen.2025.0033

Data
Data available: Yes
Data types: Data (not involving human participants), Data dictionary
How to access data: Data including cost-effectiveness models from TreeAge software will be shared upon request.
When available: With publication

Supporting Documents
Document types: None

Additional Information
Who can access the data: Anyone requesting the data.
Types of analyses: Data including cost-effectiveness models from TreeAge software will be shared upon request.
Mechanisms of data availability: with investigator support
